# The Split-Level Folding, Step-Type Tension-Relieving Suture Technique Improves the Wound Tensile Strength

**DOI:** 10.1007/s00266-025-05399-2

**Published:** 2025-11-20

**Authors:** Yige Han, Jiaqi Li, Jiaqi Liu, Yuheng Zhang, Xueyong Li, Jiezhang Tang, Baoqiang Song

**Affiliations:** 1 Department of Burns and Plastic Surgery, Tangdu Hospital, Fourth Military Medical University, No. 569, Xinsi Road, Baqiao District, Xi’an, Shanxi Province China; 2 Department of Burns and Plastic Surgery, No. 989 Hospital of the PLA Joint Logistics Support Force, Luoyang, China; 3 Department of Orthopedics, Air Force Hospital of Western Theater Command, Chengdu, China; 4 Department of Plastic Surgery, Xijing Hospital, Air Force Medical University, No. 127 Changle West Road, Xincheng District, Xi’an, Shanxi Province China

**Keywords:** Split-level folding, Step-type tension-relieving suture technique, Suture technique, Wound dehiscence, Wound healing, Wound tensile strength

## Abstract

**Background:**

Wound closure techniques are essential for skin and soft tissue repair following trauma or surgical procedures, with suturing being the most commonly employed method. Wound dehiscence is a serious postoperative complication, mostly caused by high tension and poor wound healing. The selection of an appropriate suturing method can improve the wound tensile strength and the healing process.

**Methods:**

In this study, we proposed a novel double dermal flap suturing technique, Split-Level Folding, Step-Type Tension-Relieving Suture (STS), which increases the contact area between the two sides of the sutured wound. The elliptical dorsal rat wound model was applied to compare the difference among STS, Simple Interrupted Suture (SIS) and Buried Vertical Mattress Suture (BVMS). The wound tensile strength and healing process were evaluated via mechanical testing and histological analysis at different time points after suturing.

**Results:**

On day 7 post-suturing, the ultimate load of the wound in the STS group (5.225 ± 0.661) was significantly higher than that in the SIS group (1.750 ± 0.412) and the BVMS group (3.192 ± 0.327). This advantage persisted until day 14. In histological analysis, the STS group showed superior histological scores and collagen deposition compared to the other two groups on day 7.

**Conclusion:**

The STS technique increased the dermal contact area between the two sides of the sutured wound, improved the histological healing pattern, and enhanced the wound tensile strength.

**Level of Evidence I:**

This journal requires that authors assign a level of evidence to each article. For a full description of these Evidence-Based Medicine ratings, please refer to the Table of Contents or the online Instructions to Authors www.springer.com/00266.

**Supplementary Information:**

The online version contains supplementary material available at 10.1007/s00266-025-05399-2.

## Introduction

Wound closure techniques are crucial for skin and soft tissue repair after trauma or surgery, with suturing remaining the most widely used method [1, 2]. As a serious postoperative complication, the occurrence of wound dehiscence following different surgical procedures was between 1.3 and 9.3%, with a particularly high incidence in the first 3–8 postoperative days [3–5]. Current research on preventing wound dehiscence primarily focus on factors such as pharmacological interventions, suturing technique, suture materials, wound dressings, negative pressure wound therapy [1, 6–8]. The use of an appropriate suturing technique is a relatively straightforward and cost-effective method for preventing wound dehiscence.

Simple interrupted sutures (SIS) are widely used due to their easy of operation. However, for deeper wounds, subcutaneous tissue of two sides may fail to adhere closely after SIS, potentially resulting in the formation of dead spaces and wound dehiscence [9]. Subcutaneous suturing techniques, including Buried Vertical Mattress Suture (BVMS), are commonly used for deep wounds, which can accurately align the subcutaneous layers, effectively eliminate dead space, decrease skin tension, thereby promoting wound healing and reducing wound dehiscence [10–13]. In a previous study, we provided a novel subcutaneous suturing technique on minimizing scar formation, known as the Split-Level Folding, Step-Type Tension-Relieving Suture (STS). In the STS, the dermal layer on one side of the wound is dissected, the epidermis on the contralateral side is partially excised, and the remaining dermal layer is inserted into the space beneath the dissected dermal layer on the initial side, followed by subcutaneous suturing [14]. The STS could not only decrease the dead space, optimize tension distribution, but also increase the healing contact area between dermal layers of the wound edges, which might provide better wound tensile strength.

In this study, we evaluated the wound tensile strength and healing process of the wounds sutured with STS at different time points, through mechanical testing and histological analysis in an elliptical dorsal rat wound model.

## Materials and Methods

### Animals

In this study, 48 healthy male Sprague-Dawley rats, weighing 400–500 g, were purchased from the Animal Experimental Center of the Fourth Military Medical University. Three rats were used to measure the tensile strength of normal skin, and the other 45 rats were randomly divided into three groups, with 15 rats in each group (SIS group, BVMS group and STS group), for subsequent treatment with different suturing techniques. The rats were individually housed in appropriately sized cages, maintained under a 12-hour light-dark cycle, and provided with ad libitum access to water and food. All animals were acclimatized for at least seven days prior to the experiment. The animals were fasted overnight before surgery, but water was provided ad libitum. The experiments were approved by the medical ethics committee of the Second Affiliated Hospital of Air Force Medical University (No. 20240251).

### Incision and Suturing

The anesthesia was induced using a small-animal gas inhalation anesthesia machine (ZR-02, Shanghai Puxin Instrument Technology Co., Ltd.) with an induction gas flow of 2 L/min and isoflurane concentration of 4%, followed by maintenance anesthesia with a gas flow of 2 L/min and isoflurane concentration of 1.5–2%. The dorsal area of the rats in all three groups was shaved and disinfected. Subsequently, a 3 × 1 cm elliptical full-thickness wound was created on the dorsal area of each rat. The elliptical incision was designed to simulate the high-tension wound status.

**Fig. 1:**
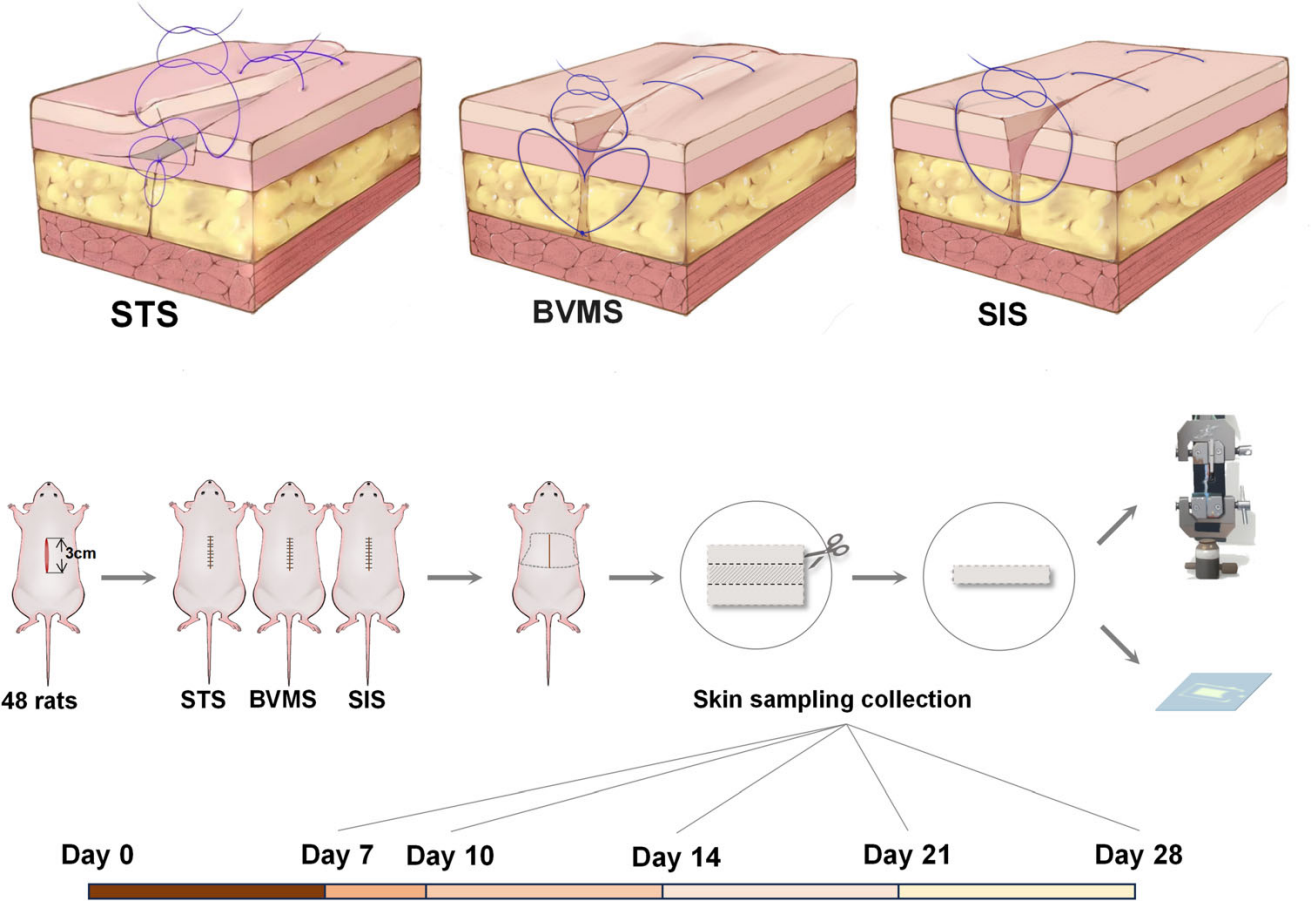
Schematic diagram of three suture techniques and experimental procedure. (above) Diagram of the three suture techniques (from left to right): Split-Level Folding, Step-Type Tension-Relieving Suture (STS), Buried Vertical Mattress Suture (BVMS), and Simple Interrupted Suture (SIS). (below) Diagram of the experimental procedure depicted experimental groups, sample collecting time points and experimental methods

Simple interrupted suture, Buried Vertical Mattress Suture and Split-Level Folding, Step-Type Tension-Relieving Suture were conducted, respectively, in SIS group, BVMS group and STS group. The details of STS (Fig. 1, above) are as follows: A subcutaneous lacuna with a depth around 3–5 mm was separated on one side of the incision (partial dermis remained on the separated subcutaneous tissue). Simultaneously, a 3–5 mm wide epidermis (and partial dermis) was excised from the contralateral side. The remaining dermis was then carefully pulled to the distal subcutaneous endpoint on the initial side, subsequently sutured to the subcutaneous tissue on both sides, encompassing a section of the dermis as well. Then the loose skin from the initial side was carefully positioned to cover the exposed dermal area. After trimming the edges of the epidermis and carefully apposing the wound, an intradermal suture was conducted. Finally, the epidermis was sutured appropriately. For the subcutaneous suturing, 5-0 PDS sutures were used; 5-0 PDS sutures were used close intracutaneous suturing; 6-0 nylon sutures were used for the epidermal suturing. The wound edges were measured before suturing with digital electronic caliper to calculate the contact area between the two sides of the wound.

After surgery, the rats were housed individually in cages to prevent wound from damage. All epidermal sutures were removed on postoperative day 7.

### Tensile Strength Testing

As shown in Fig. 1, below, a 1 cm wide rectangular full-thickness skin strip was collected from the center of the dorsal incision of the rats on postoperative days 7, 10, 14, 21, and 28. The actual width of the samples was measured and recorded using a digital electronic caliper. The skin strips were collected and stored in phosphate-buffered saline at 4 °C for subsequent mechanical testing. The ultimate load of the healing skin was assessed using a tabletop electronic testing machine (EZ-TEST 500N, Shimadzu Corporation). The skin strips were fixed using an appropriate grip and flattened under a preload. The incision was oriented perpendicular to the loading direction, positioned at the center of the template. The samples were attached to the micro-force testing system via clamps preloaded with 0.05 N. The tensile tests were performed at a displacement rate of 10 mm/min at room temperature. Force and displacement data were recorded at a sampling rate of 20 Hz, and the maximum fracture strength was recorded.

### Histological Analysis

On days 7 and 14, wound skin samples were collected, and were fixed in 4% paraformaldehyde. The tissues were embedded in paraffin blocks and sectioned into paraffin sections. The sections were stained with Hematoxylin and Eosin (H&E) staining, and Masson’s trichrome staining. Images were captured using a microscope.

The recovery of the wound was scored using a standard histological scoring system [15] based on H&E staining, Tables S1 and S2 were used as the scoring criteria for days 7 and 14, respectively. Collagen deposition was analyzed in the Masson-stained sections using ImageJ software (NIH, ImageJ 1.8, USA).

### Statistical Analysis

All statistical analyses in this study were performed using GraphPad Prism software (version 5.0; La Jolla, California, USA). Data are expressed as means ± standard deviation. One-way analysis of variance (ANOVA) was used for comparisons between multiple groups. Differences were considered statistically significant when *P* < 0.05.

## Results

### The Split-Level Folding, Step-Type Tension-Relieving Suture Increases the Contact Area Between the Two Sides of the Sutured Wound

**Fig. 2:**
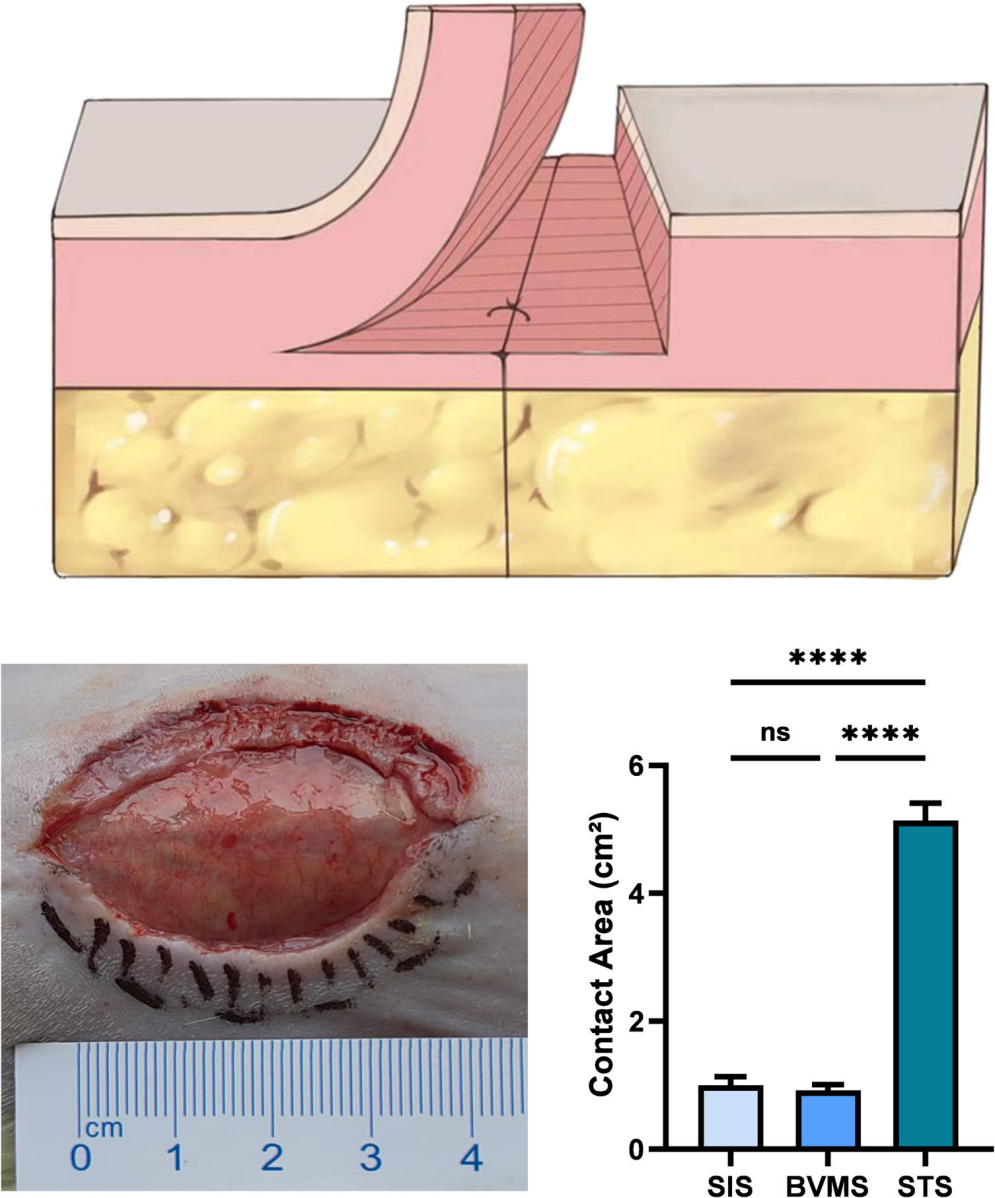
The contact area between the two sides of the sutured wound. (above) Schematic diagram of the contact area between the two sides of the sutured wound in the STS group. (below, left) Photograph of the elliptical dorsal rat wound before suturing in the STS group. (below, right) Statistical graph of the contact area in three different suture groups. (* *P* < 0.05; ** *P* < 0.01; *** *P* < 0.001; **** *P* < 0.0001)

As shown in Fig. 2 above, the contact area between the two sides of the sutured wound in the STS group is noticeably increased, due to the increase of dermal contact area. Figure 2, below, left presents a status of STS group before suturing. According to statistics, the contact area between the two sides of the sutured wound in the STS group (5.14 ± 0.27) was significantly greater than that in the BVMS group (0.92 ± 0.09) and the SIS group (0.99 ± 0.13) (Fig. 2, below, right). In the SIS group, the use of a single-layer suturing technique may lead to the formation of dead space in the subcutaneous layer, resulting in an actual contact area that is smaller than the measured area.

### The Split-Level Folding, Step-Type Tension-Relieving Suture Enhances Wound Tensile Strength During the Early Stage of Wound Healing

**Fig. 3:**
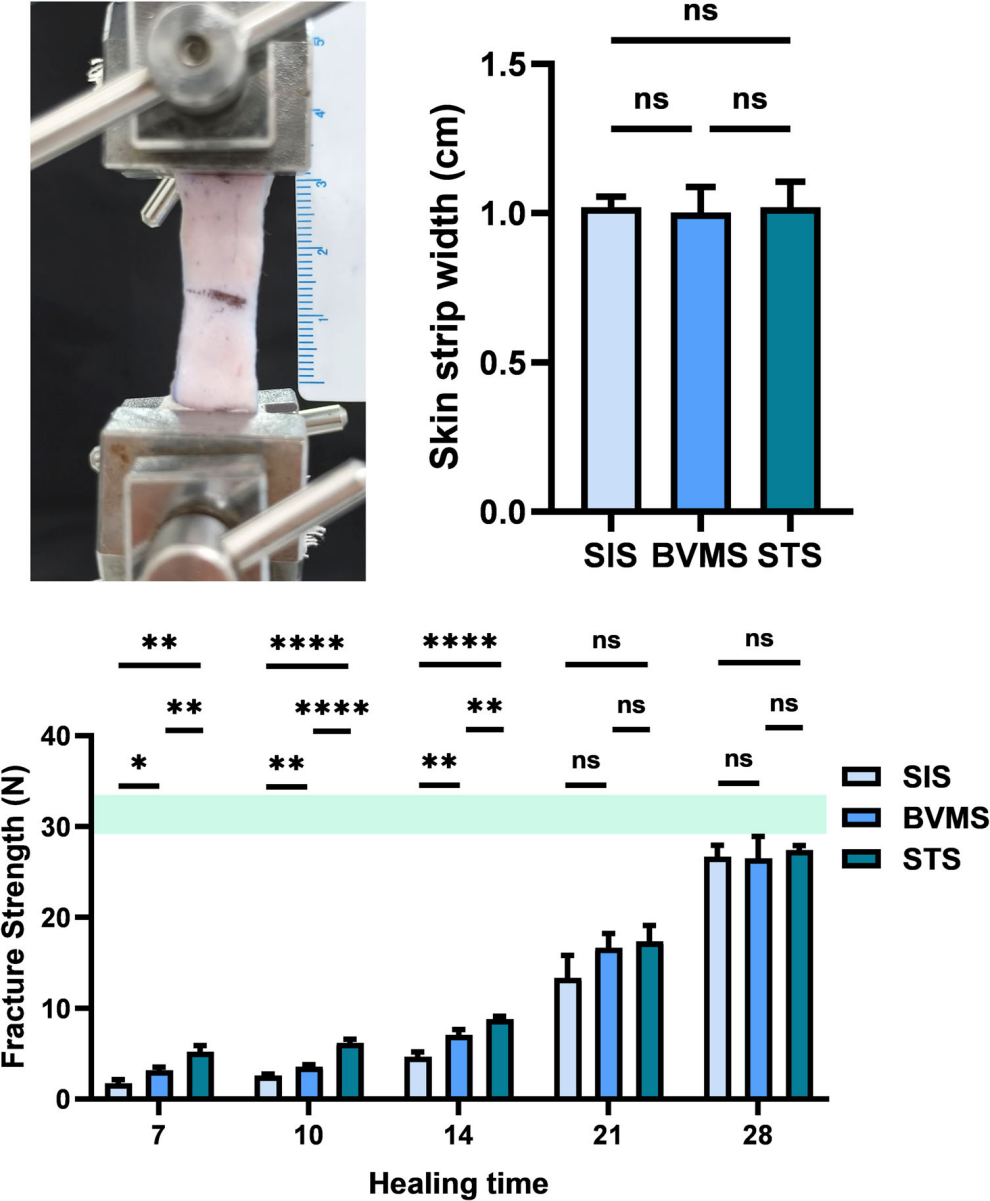
Mechanical testing of the skin strips. (above, left) Photograph of the ultimate load testing of skin strips using a tabletop electronic testing machine. (above, right) Statistical graph of the widths of the skin strips. (below) Statistical analysis of the ultimate load of the skin strips on postoperative days 7, 10, 14, 21, and 28. The green rectangular area represents the ultimate load of normal rats skin. (* *P* < 0.05; ** *P* < 0.01; *** *P* < 0.001; **** *P* < 0.0001)

In the early stage of wound healing, the STS group showed a significant advantage in wound tensile strength. The ultimate load of the sutured skin was tested by a tensile testing machine (Fig. 3, above, left), and the width of the skin strips being tested remained consistent no significant difference (Fig. 3, above, right). The ultimate load of the normal rat skin was 23.147±0.845 N. On post-suturing day 7, the ultimate load of sutured skin in the STS group (5.225 ± 0.661 N) was significantly higher than that in the SIS group (1.750 ± 0.412 N) and the BVMS group (3.192 ± 0.327 N). This advantage persisted until day 14. Between days 14 and 28, the ultimate load of the three groups gradually converged to that of the normal rat skin (Fig. 3, below).

### The Split-Level Folding, Step-Type Tension-Relieving Suture Improves the Histological Pattern of Wound Healing

**Fig. 4:**
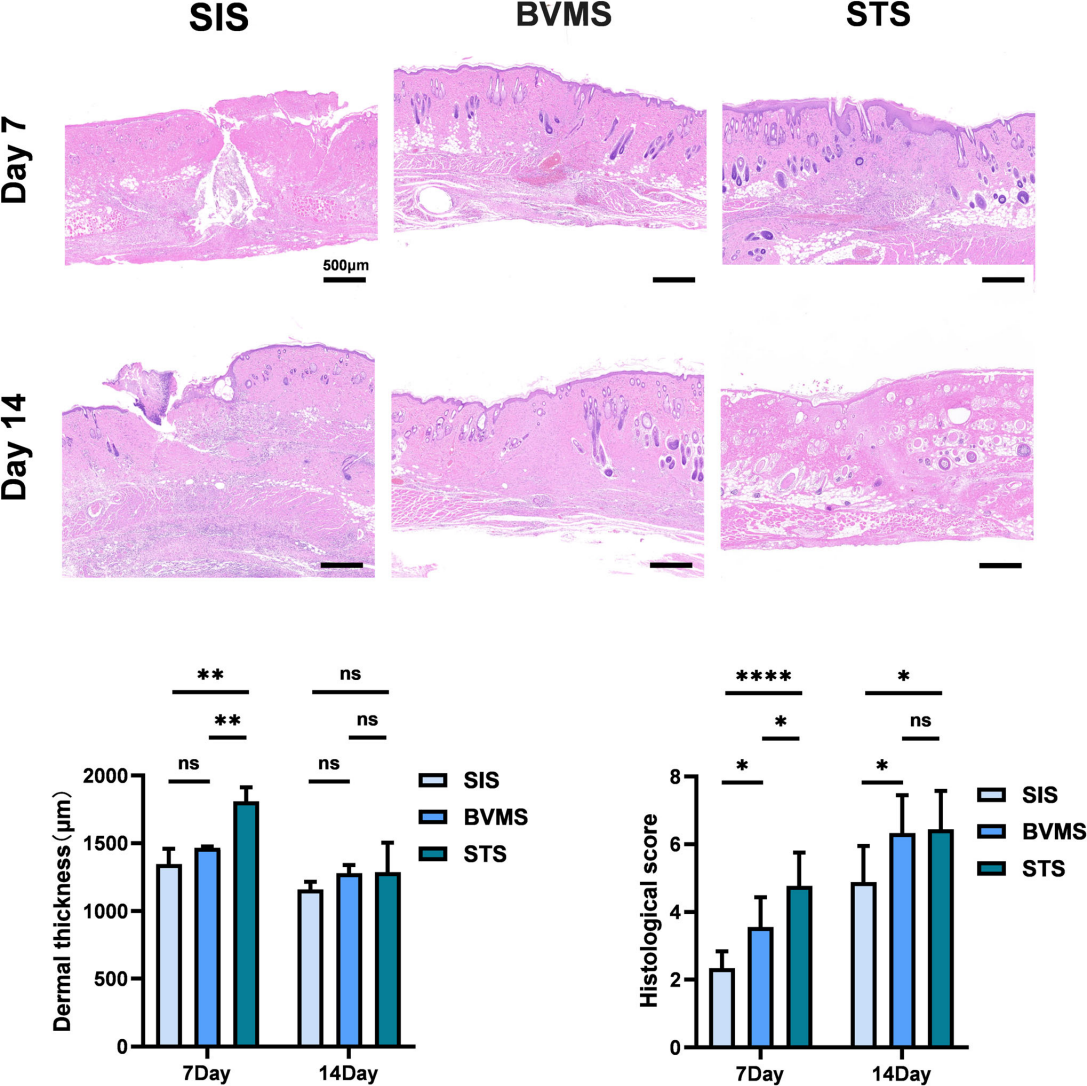
Hematoxylin and Eosin staining results of the sutured wound skins. (above) Representative HE staining images on the 7th and 14th day after operation. (below, left) Statistical graph of dermal thickness. (below, right) Statistical graph of standardized histological scoring on days 7 and 14 post-operation. (* *P* < 0.05; ** *P* < 0.01; *** *P* < 0.001; **** *P* < 0.0001)

On day 7, residual dead space was still observed in the SIS group, while the BVMS group exhibited subcutaneous hemorrhage, and both groups showed poorer epithelialization compared to the STS group (Fig. 4, above). The dermal thickness in the STS group (1808.50 ± 103.42 μm) was significantly higher than SIS (1348.23 ± 110.38 μm) and BVMS groups (1465.03 ± 11.075 μm). Moreover, the SIS group demonstrated better granulation tissue formation, increased angiogenesis (indicated by black arrows), and significantly higher histological score (4.78 ± 0.97) compared to the BVMS group (3.56 ± 0.88) and the STS group (2.33 ± 0.50) (Fig. 4, below, right).

On day 14, the granulation tissue in the two subcutaneous suturing groups (BVMS and STS) had reached a more mature stage compared to the SIS group, characterized by a reduction in cellular density and inflammation resolving (Fig. 4, above). The dermal thickness in the STS group (1287.43 ± 216.27 μm) showed no significant difference compared to BVMS group (1280.27 ± 60.03 μm). The STS group demonstrated a greater presence of skin appendages in the dermal layer. Histological score of STS group (6.44±1.13) showed no significant difference compared to the BVMS group (6.33±1.12), while the SIS group (4.89±1.05) exhibited the significantly lowest histological score. (Fig. 4, below, right).

In addition, dermal thickness in all three groups was measured and statistically analyzed by ImageJ. On day 7, the dermal thickness of the STS group was greater than that of the other two groups. On day 14, no significant differences were observed among the three groups (Fig. 4, below, left).

**Fig. 5:**
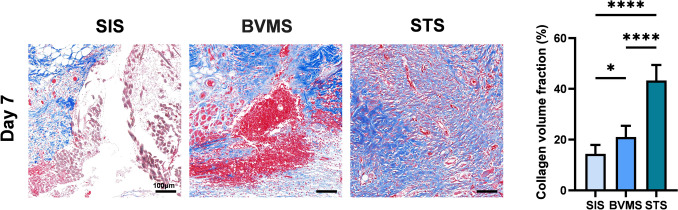
Masson’s trichrome staining of the sutured wound skins. (above) Representative Masson’s trichrome staining images on the 7th day after operation. (below) Statistical graph of collagen deposition on the 7th day after operation. (* *P* < 0.05; ** *P* < 0.01; *** *P* < 0.001; **** *P* < 0.0001)

Masson staining revealed that collagen deposition in the STS group (43.30 ± 6.12%) was significantly better than SIS group (14.44 ± 3.47%) and BVMS group (21.00 ± 4.43%) (Fig. 5). The results were consistent with the ranking of the maximum tensile strength tolerated by the three groups, greater wound tensile strength might correlate with better collagen deposition.

## Discussion

Suturing is the preferred approach for wound closure. [1] As a complication that increases the risk of pain, hemorrhage, infection, delayed healing, postoperative wound dehiscence increases hospitalization duration, healthcare costs, and readmission rates. At present, postoperative wound dehiscence remains a high incidence rate [6, 7], typically in 5–8 days after surgery (early stage of wound healing). Among various methods reducing postoperative wound dehiscence, appropriate suturing techniques could be the most cost-effective and easily applicable solution.

Previous suturing methods are typically designed to reduce wound dehiscence and promote healing by preventing wound eversion, eliminating dead space, and ensuring a close and precise approximation of the dermal and subcutaneous layers [16, 17]. Among these methods, buried vertical mattress suturing (BVMS) is widely adopted due to its excellent ability to achieve these goals [10]. It has been demonstrated that different incision closure techniques can influence the synthesis of collagen fibers near the incision area and result in varying wound tensile strength [18]. The wound tensile strength is related to the number and bonding of collagen fibers, and influenced by cytokine activity [19–21]. We speculated that during wound healing, the close alignment and increased contact area of the dermis might contribute to the promotion of more cytokine activity, the facilitation of cellular migration on both sides of the wound, and the enhancement of collagen deposition, thereby increasing the wound tensile strength and accelerating the healing process. Therefore, we propose a novel suturing technique, the Split-Level Folding Step-Type Tension-Relieving Suture Technique (STS), in which the contact area between the two edges of the sutured wound is noticeably increased, because of the enlargement of dermal contact area. [14].

In this study, we assessed the wound tensile strength of wound skin closed by different suturing methods, and found that within 14 days post-surgery, the STS group exhibited the highest wound tensile strength, followed by the BVMS group. The results indicated that STS improved wound healing quality in the early stages and demonstrated its excellent resistance to rupture. However, between days 14 and 28, the tensile strength advantage of the STS group gradually diminished compared to the other two groups.

For full-thickness skin wounds, healing is a complex physiological process. Wound healing involves several cellular and molecular mechanisms and consists of hemostasis, inflammation, proliferation, remodeling phases [22]. The advantage of wound tensile strength in the STS group mainly existed within 14 days post-suturing. As a result, hematoxylin and eosin staining was applied to evaluate the wound healing quality on days 7 and 14. On day 7, compared to the SIS group, which still had dead space, and the BVMS group, which had subcutaneous hematoma and thin epidermis, the STS group showed the greatest histological advantage, including the best angiogenesis and abundant granulation tissue formation. On day 14, the histological scores of the two subdermal suturing groups presented no significant difference, but more skin appendages and fewer inflammatory cells existed in the STS group. The larger dermal contact area might facilitate cell migration and angiogenesis in the early stages of wound healing, and promotes the resolution of inflammation and the restoration of skin appendages in the later stages. The Step-Type Suture technique accelerated the healing process and contributed to higher-quality wound repair.

In Masson staining, SIS group showed the greatest collagen deposition on day 7. A larger contact area in the STS group is likely to facilitate the migration of fibroblasts and the exchange of cytokines, thereby promoting the production of collagen fibers at the incision site and enhancing the tensile strength of the wound [19].

Although the histological and biomechanical effects of the STS technique on promoting wound healing and preventing wound dehiscence have been validated, several disadvantages of study existed. The study lacks quantitative relationship between the wound tensile strength and the contact area between the two sides of the sutured wound. Moreover, the related molecular mechanisms require further investigation. In addition, the effects of STS on improving wound tensile strength need pre-clinical validation through large animal experiments. These disadvantages provide promising directions for future research.

## Conclusions

The Split-Level Folding, Step-Type Tension-Relieving Suture enhanced wound tensile strength by significantly increasing the contact area between the two sides of the sutured wound. Skin wounds sutured with STS showed superior angiogenesis, granulation tissue formation, re-epithelialization and collagen deposition. As a cost-effective, easy operating suture method, STS has considerable potential for reducing wound dehiscence in clinical practice.

## Supplementary Information

Below is the link to the electronic supplementary material.

Supplementary file1 (DOCX 16 kb)
