# Supplementary material for: The Split-Level Folding, Step-Type Tension-Relieving Suture Technique Improves the Wound Tensile Strength
Source: Aesthetic Plast Surg. 2025 Nov 20;50(1):407–14. doi: 10.1007/s00266-025-05399-2 (PMC12916890; doi:10.1007/s00266-025-05399-2)
Supplement: Supplementary file 1 — Supplementary file1 (DOCX 16 kb) [file 266_2025_5399_MOESM1_ESM.docx]

| Parameter | Description | Criteria | Score |
| --- | --- | --- | --- |
| Re-epithelization | Complete | 95–100% | 2 |
|  | Partial | <95%; >0% | 1 |
|  | None | 0% | 0 |
| Epidermal thickness index (Recommendation: only assess if Re-epithelization = complete) | Normal | 95–105% | 2 |
|  | Hypertrophy | >105% | 1 |
|  | Hypoplasia | <95% | 0 |
| Keratinization visual inspection (Recommendation: only assess if re-epithelization = complete) | Yes | Loosely attached/lost layers OR thick parakeratotic stratum corneum | 2 |
|  | No | None | 0 |

**Table S1.** Histological Scoring System for Wound Tissue Samples at 7 Days.

**Table S2.** Histological Scoring System for Wound Tissue Samples at 14 Days.

| Parameter | Description | Criteria | Score |
| --- | --- | --- | --- |
| Re-epithelization | Complete | 95–100% | 2 |
|  | Partial | <95%; >0% | 1 |
|  | None | 0% | 0 |
| Epidermal thickness index (Recommendation: only assess if Re-epithelization = complete) | Normal | 95–105% | 2 |
|  | Hypertrophy | >105% | 1 |
|  | Hypoplasia | <95% | 0 |
| Keratinization visual inspection (Recommendation: only assess if re-epithelization = complete) | Yes | Loosely attached/lost layers OR thick parakeratotic stratum corneum | 2 |
|  | No | None | 0 |
| Granulation tissue Visual inspection and Absolute measure (μm) | Intact dermis | Dermal layer intact no granular infiltrates consistent with healing | 2 |
|  | Thick Granulation tissue | >100 μm | 1 |
|  | Thin Granulation tissue | <100 μm | 0 |
